# Sparse Solution of Fiber Orientation Distribution Function by Diffusion Decomposition

**DOI:** 10.1371/journal.pone.0075747

**Published:** 2013-10-11

**Authors:** Fang-Cheng Yeh, Wen-Yih Isaac Tseng

**Affiliations:** 1 Department of Biomedical Engineering, Carnegie Mellon University, Pittsburgh, Pennsylvania, United States of America; 2 Center for Optoelectronic Medicine, National Taiwan University College of Medicine, Taipei, Taiwan; 3 Department of Medical Imaging, National Taiwan University Hospital, Taipei, Taiwan; Cuban Neuroscience Center, Cuba

## Abstract

Fiber orientation is the key information in diffusion tractography. Several deconvolution methods have been proposed to obtain fiber orientations by estimating a fiber orientation distribution function (ODF). However, the *L*_2_ regularization used in deconvolution often leads to false fibers that compromise the specificity of the results. To address this problem, we propose a method called diffusion decomposition, which obtains a sparse solution of fiber ODF by decomposing the diffusion ODF obtained from q-ball imaging (QBI), diffusion spectrum imaging (DSI), or generalized q-sampling imaging (GQI). A simulation study, a phantom study, and an *in-vivo* study were conducted to examine the performance of diffusion decomposition. The simulation study showed that diffusion decomposition was more accurate than both constrained spherical deconvolution and ball-and-sticks model. The phantom study showed that the angular error of diffusion decomposition was significantly lower than those of constrained spherical deconvolution at 30° crossing and ball-and-sticks model at 60° crossing. The *in-vivo* study showed that diffusion decomposition can be applied to QBI, DSI, or GQI, and the resolved fiber orientations were consistent regardless of the diffusion sampling schemes and diffusion reconstruction methods. The performance of diffusion decomposition was further demonstrated by resolving crossing fibers on a 30-direction QBI dataset and a 40-direction DSI dataset. In conclusion, diffusion decomposition can improve angular resolution and resolve crossing fibers in datasets with low SNR and substantially reduced number of diffusion encoding directions. These advantages may be valuable for human connectome studies and clinical research.

## Introduction

**Figure 1:**
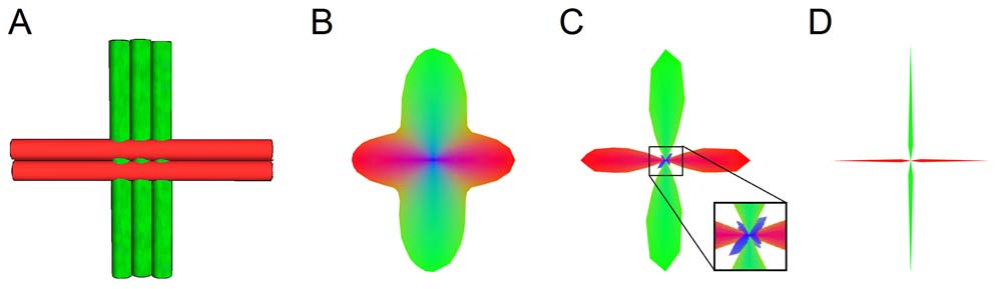
An example of two crossing fibers at right angles. (A) The layout of the crossing fibers. (B) The corresponding diffusion ODF, showing the orientation distribution of the water diffusion due to the right angle crossing environment. (C) The corresponding fiber ODF obtained from diffusion deconvolution has non-zero values in most of the orientations, as shown in the blue colored fluctuation around the origin. This baseline fluctuation often causes false identification of crossing fibers. (D) The actual fiber ODF has zero values at most of the orientations and non-zero values only at the fiber orientations, suggesting that the fiber ODF is sparse.

Crossing fiber problem is still under active research in the field of diffusion MRI, and a method that offers accurate fiber orientation is the cornerstone of human connectome studies since it can facilitate fiber tracking and provide better mapping of neuronal connections [1], [2]. To resolve crossing fibers, methods that make use of the orientation distribution function (ODF) have been widely used [3]. Tuch [4] proposed q-ball imaging (QBI) to estimate the diffusion ODF (dODF) by applying Funk-Radon transform to high angular resolution diffusion images (HARDI) [5]. Wedeen et al. [6] proposed diffusion spectrum imaging (DSI), which obtains dODF by applying inverse Fourier transform to diffusion MR signals and calculating the second moment along the radial distribution of the transformed signals. To further extend the applicability, Yeh et al. [7] proposed generalized q-sampling imaging (GQI), which can be applied to a variety of diffusion sampling schemes to obtain dODFs, and the results are consistent with those from QBI and DSI. Although these dODF methods have been used to determine fiber orientations, their accuracy is limited by the blurred contour of dODF. This problem is demonstrated in Fig. 1, an example of two fiber populations crossed at right angles (Fig. 1A). The fiber orientations can be resolved by the peak orientations of the dODF (Fig. 1B), but the blurred contour of the dODF may fail to resolve crossing fibers if the crossing angle is sufficiently small.

To better characterize fiber distribution, studies have used spherical deconvolution to obtain fiber ODF (fODF) and to measure the orientation distribution of fiber volume fractions [8], [9]. Spherical deconvolution estimated the signal pattern of a single fiber bundle and applied deconvolution to the spherical harmonics of diffusion MR signals to calculate fODF. An example of fODF is shown in Fig. 1C, where fODF presents a contour sharper than dODF’s and achieves better angular resolution. This advantage makes fODF a useful way to resolve crossing fibers for diffusion MRI fiber tracking. Further studies have proposed improved methods to address the problem of partial volume effect [10], [11], which can corrupt fODFs and produce false peaks. Moreover, different computational approaches have been proposed to sharpen fODFs, enforce non-negativity, or achieve more robust fODFs estimation [12], [13], [14], [15], [16], [17]. Although fODF has been shown to be sensitive to crossing fibers, its specificity has been called into question [11], [18]. fODF often has baseline fluctuation that may give rise to false peaks, as illustrated in the zoom-in figure of Fig. 1C. This fluctuation problem is often handled by applying an ODF filter, but the filter can indiscriminately eliminate less salient fibers and decrease the sensitivity of fODF. Besides the baseline fluctuation problem, *L*_2_ regularization also introduces blurring to fODF; this is in contrast to the true fODF (Fig. 1D), which has non-zero distribution value at fiber orientations and zeros elsewhere—a feature known as sparsity. The sparsity feature of fODF has inspired studies to make use of *L_1_* regularization to promote sparsity and to provide a robust estimation for fODF [19], [20].

In this study, we extended the *L_1_* regularization paradigm to dODFs obtained from QBI, DSI, or GQI, aiming to get a sparse solution of fODF and to provide better resolving power for crossing fibers. The diffusion model was formulated as a regression situation. We used dODF as the response and component dODFs as the regressors. The coefficients of the regression equation comprised the fODF and were estimated by a popular estimation approach called least absolute shrinkage and selection operator (LASSO) [21], which used *L*_1_ regularization to promote sparsity of the solution. Although several LASSO estimation algorithms have been proposed [20], [22], we adopted one of the simplest approaches, the forward stagewise method [23], and modified it to consider the nonnegative constraints of fODF. The resulting algorithm, coined diffusion decomposition, recursively decomposed a dODF to obtain the sparse solution of fODF.

To examine the performance of diffusion decomposition, we conducted a simulation and a phantom study using the HARDI scheme. The results were compared with constrained spherical deconvolution (CSD) [24] and ball-and-sticks model [25]. In our *in-vivo* study, we applied diffusion decomposition to dODFs obtained from QBI, DSI, or GQI. The performance was compared with a corresponding deconvolution approach called diffusion deconvolution [11], which is not limited to HARDI scheme and can be equally applied to QBI, DSI, and GQI. A sensitivity and specificity test was conducted to examine the performance of diffusion decomposition on datasets with reduced number of diffusion encoding directions. Lastly, the mapping of the fiber volume fraction derived from diffusion decomposition was compared to the fractional anisotropy (FA) obtained from DTI analysis.

## Materials and Methods

### Diffusion model

We modeled a dODF using the mixed diffusion model [11]: a dODF can be viewed as a linear combination of multiple dODF components, meaning that the diffusion signal of a dODF is the summation of the diffusion signals from each constituted fiber components. Specifically, the overall dODF 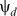 is composed of a background isotropic component *f*_0_ *e* and a series of component dODFs, 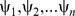, each of which represents a fiber population oriented at a unique direction.
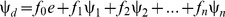
(1)


where *f*_0_ is the volume fraction of the background isotropic diffusion, and *e* is an all-one vector modeling the isotropic diffusion. The coefficients *f*_1_, *f*_2_, …*f*_n_ are the fiber volume fractions for each of the component dODFs. In our implementation, we further scaled the sum of the volume fractions (i.e.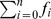) to the spin density because the total volume is in fact proportional to the amount of the spins. The same scaling was also used in diffusion deconvolution to avoid the partial volume effect from the gray matter [11]. Furthermore, each dODF in Eq. (1) was represented by a vector with finite dimensions. Specifically, the sampling orientations of a dODF were defined by the vertices of an 8-fold tessellated icosahedron, which has a total number of 642 orientations. Since the dODF is assumed to be symmetric about the origin, a dODF can be represented by a 321-dimension vector, resulting in an angular resolution of around 8°. These 321 unique orientations were also used to assign the fiber orientations of the component dODFs, resulting in 321 component dODFs (*n* = 321). Using this setting, the orientation distribution of the volume fractions, *f*_1_, *f*_2_, …*f*_n_, constitute the fODF that we aimed to estimate.

To solve Eq. (1), we first estimated the dODF, 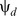, using standard procedures of QBI, DSI, or GQI, and modeled the component dODFs with a putative common characteristic dODF that described the diffusion profile of a single fiber population. We selected the characteristic dODF from the voxel having the highest anisotropy, a strategy similar to deconvolution methods [8], [26]. To calculate each component dODF from the characteristic dODF, we smoothed the characteristic dODF as described in our previous work [11]. The following is the formula for estimating the *i*-th component dODF:



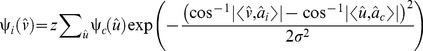
(2)


where 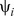 is the *i*-th component dODF, and 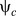 is the common characteristic dODF. 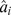 and 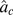 are the fiber orientations of 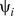 and 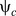, respectively. The operator 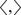 calculates the inner product of two vectors, σ is the standard deviation for the Gaussian radial basis kernel, and *z* is a normalizing factor to ensure that the component dODF integrates to one. The summation iterates through all sampling directions, 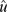, and σ  =  9° was used as that in our previous work.

### LASSO estimator

Without loss of generality, the regression equation in Eq. (1) can be reformulated by normalizing the vector of each component dODF in a way that the mean is zero, and the length of the vector is one.
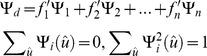
(3)


One should note that the isotropic component is removed since the mean is shifted to zero, and the coefficients are rescaled since the corresponding component dODFs are normalized. The sparse solution for the coefficients (i.e. the scaled volume fractions) can be estimated by the LASSO estimator, which uses *L*_1_ regularization to promote sparsity.
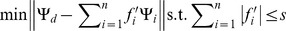
(4)


The regularization is controlled by *s*, and it has been shown that the solutions for different levels of *s* can be obtained by iteratively including different regressors (component dODFs) in the solution set [10], [22], [23]. This leads to our diffusion decomposition algorithm, which is based on the forward stagewise method [23] and further considers the nonnegative constraint of fODF.

### Diffusion decomposition

Table 1 shows the diffusion decomposition algorithm, which includes the following steps (the C++ source codes of diffusion decomposition are available to the public at http://dsi-studio.labsolver.org). First of all, *selection*, the algorithm finds the component dODF that is most correlated with the dODF.

**Table 1:** Diffusion Decomposition Algorithm.

Input	Comments
ψ*_d_*	dODF
ψ_ 1_, ψ_2_, … ψ*_n_*	Component dODF
Ψ*_d_*	Normalized dODF^a^
Ψ_1_, Ψ_2_, … Ψ*_n_*	Normalized component dODF
*ε*	Decomposition fraction
**Output**	**Comments**
*f*_0_	Volume fraction of the isotropic background diffusion
*f*_1_, *f*_2_, …*f_n_*	Volume fractions of the component dODFs
**Algorithm**	**Comments**
1. while 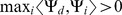 and |A| < *m*	Repeat 1.1, 1.2, and 1.3 as long as the condition holds.
1.1. 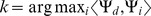	Selection: Select the most correlated component dODF
1.2. 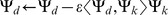	Decomposition: Decompose a small volume from the dODF
1.3. A←A 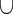 *k*	Record the selected component dODFs
2. 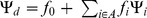	Solve volume fractions by ordinary least squares^b^
3. If min*_i_* (*f_i_*)<0 then A←A \ argmin*_i_* (*f_i_*)and go to 2	If any of the volume fractions is negative, set the most negative one to zero and remove it from set *A* and solve the regression again.
4. If *f*_0_ < 0 then *f*_0_ ← 0 and go to 2	If the volume fraction of background diffusion is negative, force it to zero and solve the regression again.

a The normalized dODF is calculated by 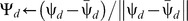.

b *f_i_* ← 0 if 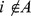.



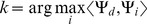
(5)where 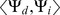 is the inner product of the normalized dODF and a normalized component dODF. One should note that in Eq. (5) diffusion decomposition considers only the positive correlation due to the nonnegative constraint of the solution. The next step is *decomposition*, which removes a small volume of Ψ*_k_* from Ψ*_d_*:
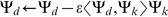
(6)


where ε is the decomposition fraction. These two steps, *selection* and *decomposition*, are recursively iterated until the maximum correlation is less than or equal to zero, or a total of *m* unique component dODFs are selected. The selected component dODFs in the *selection-decomposition* recursion are recorded in a component set, A, whereas those not selected have zero fiber volume fractions. One should note that the same component dODF can be selected more than once, but the component set only includes unique elements. After the *selection-decomposition* recursion, the algorithm proceeds to *estimation*, in which we reformulated Eq. (1) as follow.



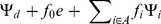
(7)


One should note that in Eq. 7 the original dODFs are used instead of the normalized dODFs, and the solution for the volume fractions can be obtained by ordinary linear regression. To enforce the nonnegative constraint, if any of the volume fractions is negative, the most negative volume fraction is forced to zero, and then the equation is solved again until all volume fractions are nonnegative. The resulting fiber volume fractions *f*_1_, *f*_2_, …*f*_n_ constitute the solution of the fODF.

### Parameter settings and computation efficiency

The computation efficiency of diffusion decomposition algorithm is mainly determined by the decomposition fraction, and it is usually set to a sufficiently small value to achieve a good estimation [22], [23]. However, this setting may burden the computation since the initial decompositions are always repeated on the same component dODF until another component dODF becomes dominant. To reduce the number of repetition, we can decompose a larger fraction that just allows the second component to dominate, as implemented in the least angle regression algorithm [22]. Following this paradigm, we can derive the algorithm for solving this large decomposition fraction. 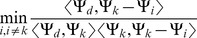
(8)


where Ψ*_k_* is the most correlated component dODF selected in the first step of the algorithm, and the minimum searches all other components dODFs (Ψ*_i_*, *i*  =  1, 2, …, *n*, *i* ≠ *k*) to find the least decomposition fraction required to allow another component dODF to dominate. In this study, we used Eq. 8 only for the first decomposition, and then the decomposition fraction was set to a fixed value thereafter (e.g. 0.05).

The maximum size of the component set, A, may also affect the computation efficiency, and a suitable value can be determined by a priori information that human brain has limited number of crossing fiber populations stacked within a voxel. For most of the white matter area, the number of crossing fibers is no more than 3 [27], [28], and in this study, we used a maximum size of 10 to get a balance between detection ability and computation time.

### Simulation study

We simulated crossing fibers to evaluate the performance of diffusion decomposition and to compare it with that of CSD and ball-and-sticks model. A multiple-tensor model was used to obtain the diffusion signals of crossing fibers. As summarized in Table 2, the model has a component of isotropic diffusion and two fiber populations [5], [29].

**Table 2:** Summary of simulation and phantom studies.

	b-value (s/mm^2^_)_	directions	SNR	crossing angles	simulation/experiment settings
Simulation 1	1500	160	6.8	18° to 90°*	Two tensors and an isotropic component
Simulation 2	1500	55	2.3	18° to 90°*	Two tensors and an isotropic component
Phantom	4000	160	4.6	30° and 60°	Two sets of capillary tubes immersed in water

* divided into 40 steps.



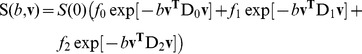
(12)where *S*(0) is the baseline signal without diffusion encoding (b0). *b* and **v** are the b-value and the unit vector of the applied diffusion gradient, respectively. *f*_0_ is the volume fraction of the isotropic background diffusion. *f*_1_ and *f*_2_ are the volume fractions of the two fiber populations. D_0_, D_1_, and D_2_ are the diffusion tensor matrices for these three diffusion components (D_0_ is a multiple of the identity matrix). The inclusion of the isotropic background diffusion results in partial volume effect, which is a more challenging condition for obtaining fODFs. We simulated this model with a variety of parameter combinations, including fiber volume fraction, crossing angle, and FA. In the simulation study, the *f*_0_ was set to 0.2, and *f*_1_ were assigned from 0.50×(1−*f*_0_) to 0.90×(1−*f*_0_), divided into 40 steps of size 0.01. The remaining volume was occupied by *f*_2_. The crossing angles ranged from 18° to 90°, divided into 40 steps of size 1.8°.FA values for both of the simulated fibers were 0.4, 0.5, 0.6, and 0.7. Two signal-to-noise (SNR) settings were simulated. The first SNR setting was simulated with Rician noise [30] added to 160 diffusion weighted images (HARDI, b-value  =  1,500 s/mm^2^) and a b0 image. The mean diffusivity was 1.0×10^−3 ^mm^2^/s. The SNR of the b0 image was 40, and the average SNR of the 160 diffusion weighted images was 6.8. The second SNR setting was simulated with Rician noise added to 55 diffusion weighted images (HARDI, b-value  =  1,500 s/mm^2^) and a b0 image. The mean diffusivity was 0.5×10^−3 ^mm^2^/s. The SNR of the b0 image was 20, and the average SNR of the 55 diffusion weighted images was 2.3. To average the angular deviation, each parameter combination (fiber volume fractions, crossing angle, and FA) was simulated independently for a total of 100 trials. The source codes for generating the simulation images are publicly available at https://github.com/frankyeh/DSI-Studio.

MRtrix (http://www.nitrc.org/projects/mrtrix/) was used to conduct CSD. To ensure the best performance, four different response functions were estimated for each FA value simulation (0.4, 0.5, 0.6, and 0.7). The estimated response functions were inspected to confirm the accuracy as recommended by the MRtrix documentation. A harmonic order of 6 was used (lmax  =  6), which was lower than the default setting. We did not use the default value because it failed to generate a valid disk-shaped response function and resulted in fODFs with meaningless spikes. The peak orientations were calculated using the peak finding tool provided in the MRtrix package.

The ball-and-sticks model was calculated using the bedpostx program in the FMRIB’s Diffusion Toolkit v2.0 package (http://fsl.fmrib.ox.ac.uk/). Rician noise model was used, and the maximum number of fibers was set to 2 to match the simulation setting.

For each comparing method, the angular error was calculated as follows. If a method resolved more than two fiber orientations (a common scenario in deconvolution), the most prominent two orientations were used whereas the rest were discarded. If a method resolved only one fiber orientation, it was regarded as resolving two fibers at the same orientation. For each voxel, the two resolved fiber orientations were compared with the ground truth to calculate the angular deviation. The performance was reported by the average of these two deviation values. Although this evaluation approach may under-represent the error when too few or too many fiber directions are identified, the results are still valid as long as it is not biased toward one method over another.

### Phantom study

The diffusion phantom was created by using silica capillary tubes with an inner/outer diameter of 20/90 μm (Polymicro Technologies, Phoenix, Arizona, USA. These tubes were aligned in two placeholders, and the relative orientations were set to 30° and 60° to simulate crossing fiber patterns. For each crossing angle setting, one placeholder was placed vertically and another one crossed it at the assigned crossing angle. The phantom was scanned in a 9.4 Tesla Bruker spectrometer (Bruker Companies, Ettlingen, Germany) with 2D-FT stimulated-echo diffusion-weighted imaging with TR/TE  =  1900/13.8 ms, matrix size  =  32×32, FOV  =  25 mm×25mm, slice thickness  =  3.6 mm, number of diffusion sampling directions  =  160, b-value  =  4000 s/mm^2^, and NEX  =  4. T_2_-weighted images were also acquired to measure the exact crossing angles of the phantom. For crossing angles originally arranged at 30° and 60°, the measured crossing angles on the T_2_-weighted images were 31.48° and 63.59°, respectively. The angle was measured by the crossing angles of placeholders on the T_2_-weighted images. Similar to the simulation study, CSD, ball-and-sticks model, and diffusion decomposition were conducted using the same parameter settings. The angular deviation was calculated using the resolved fiber orientations and the actual fiber orientations shown on the T_2_-weighted images.

### In-vivo study

Diffusion MRI was acquired from a 25-year-old healthy male participant on a 3T MRI system (TIM Trio, Siemens, Erlangen, Germany). The scan was performed using a 12-channel head coil and a single-shot twice-refocused echo planar imaging (EPI) diffusion pulse sequence. On the same subject, shell (for QBI), grid (for DSI), and two-shell (for GQI) sampling schemes were acquired consecutively using the same spatial parameters: field of view  =  240 mm×240 mm, matrix size  =  96×96, slice thickness  =  2.5 mm (no gap), the number of the slices  =  40 covering the cerebral cortex, resulting in isotropic voxel size of 2.5 mm. The shell scheme was acquired by 252 diffusion gradient directions, b-value  =  4000 sec/mm^2^, and TR/TE  =  7200 ms/133 ms. The grid scheme was acquired by 202 diffusion gradient directions at the grid points within a sphere in the q-space, maximum b-value  =  4000 sec/mm^2^, and TR/TE  =  7200 ms/144 ms. The two-shell scheme was acquired by two DTI scans based on the built-in DTI b-table. One DTI dataset had 64 diffusion-weighted images (DWI) acquired by b-value  =  3000 sec/mm^2^, and TR/TE  =  6300 ms/121 ms. Another DTI dataset had 30 gradient directions, b-value  =  1500 sec/mm^2^, and TR/TE  =  5500 ms/101 ms. Diffusion deconvolution and diffusion decomposition were conducted using the same dataset to facilitate comparison. Diffusion deconvolution was conducted using a regularization parameter of 7, a value recommended for *in-vivo* data [11], whereas diffusion decomposition was conducted using the same setting as the phantom study.

### Ethics Statement

Data were analyzed anonymously, and written inform consent was waived because the study fulfills the following requirements. The study has the lowest risk. The risk to the studied subjects does not exceed the possible risks of people who do not participate in the study. Waiving the prior consent does not affect the rights and interests of the studied subjects. (File# 1010265083, Ministry of Health, Executive Yuan, Taiwan). The research procedures were also approved by the Institutional Review Board of National Taiwan University Hospital.

### Sensitivity and Specificity Test

We applied diffusion decomposition to datasets with reduced number of diffusion encoding directions (termed reduced datasets hereafter) and compared the results with datasets acquired by full diffusion encoding directions (termed full datasets hereafter). The performance of diffusion decomposition was quantified in terms of the angular error and was compared with that of diffusion deconvolution [11], which can be equally applied to both shell and grid sampling schemes for comparison. For the shell scheme, the reduced dataset was the 30-direction dataset acquired with b-value  =  1500 mm/s, and the full dataset was the 252-direction shell dataset acquired with b-value  =  4000 mm/s. For the grid scheme, the reduced dataset was the 40 lowest b-values acquisitions of the full 202-direction grid dataset (maximum b-value  =  2300 sec/mm^2^). The fODFs of the full datasets (both shell and grid) were calculated by diffusion deconvolution with a regularization parameter of 7, a value recommended for *in-vivo* data [11], whereas the fODFs of the reduced datasets were calculated separately by diffusion deconvolution and diffusion decomposition for comparison. Since the performance may be affected by different parameter settings, the reduced dataset was processed under 5 different parameters for each method. For diffusion decomposition, we used decomposition fractions of 0.01, 0.02, 0.05, 0.1, and 0.2. For diffusion deconvolution, the regularization parameters were set to 1, 2, 4, 8, and 16. For both reconstructions, the fiber orientations were determined by peak orientations on the fODFs.

To calculate the angular error related to sensitivity, we selected each fiber in the full dataset and calculated its angular error with respect to a corresponding fiber in the reduced dataset. If multiple fibers were resolved in the reduced dataset, the one that had the least angular error was chosen. If no fiber was resolved, an angular error of 45° (the expected angular error if the fiber was resolved at a random orientation) was assigned. Specificity was examined conversely. We selected each fiber in the reduced dataset and calculated its angular error with respect to a corresponding fiber in the full dataset. If multiple fibers were resolved in the full dataset, the one that had the least angular error was chosen. If no fiber was resolved, an angular error of 45° was assigned. To exclude gray matter in the analysis, the angular error was calculated by fibers in white matter area defined by applying a threshold to the quantitative anisotropy (QA) [7] of the full dataset. The exact threshold values were adjusted by comparing the extent of the threshold with the gray-white matter junction to achieve the best match of the coverage in the white matter.

## Results

### Simulation study

**Figure 2:**
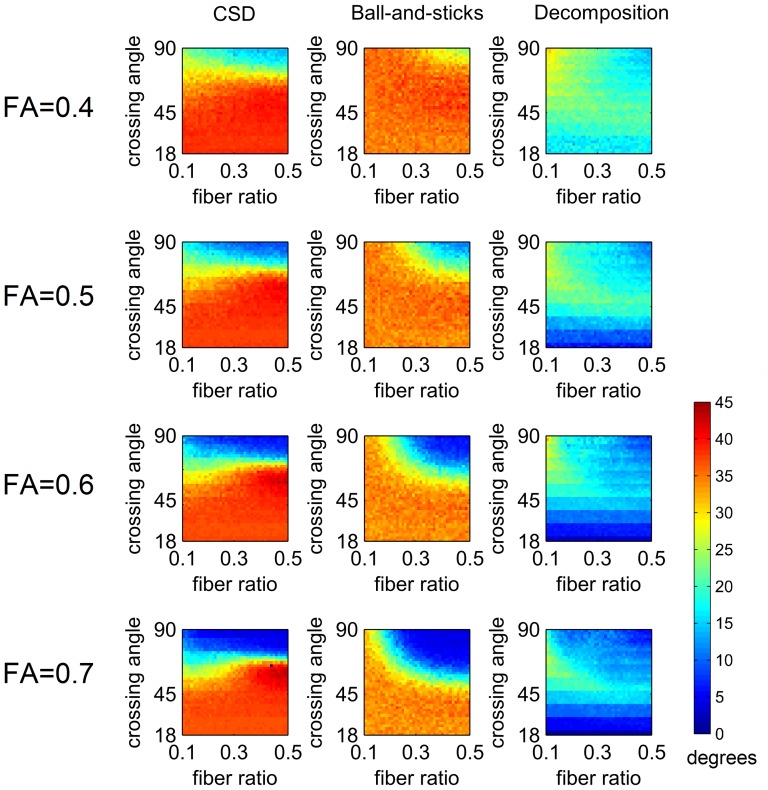
The average angular deviation of constrained spherical deconvolution (CSD), ball-and-sticks model, and diffusion decomposition applied to 160 diffusion weighted images with SNR of 6.8. The performance is examined under different fractional anisotropy, crossing angles, and fiber volume fractions. Diffusion decomposition outperforms CSD and ball-and-sticks model in smaller crossing angles.

Figure 2 shows the average angular deviation of CSD, ball-and-sticks model, and diffusion decomposition applied to 160 diffusion weighted images with SNR of 6.8. All methods were applied to different combinations of crossing angle, fiber volume fractions, and FA. As shown in the figure, CSD performs well with crossing fiber greater than 45°, but its angular deviation increases in smaller crossing angles due to the false peaks in fODFs. This result demonstrates a typical specificity problem of CSD in smaller crossing angles. The noise in the diffusion signals can give rise to false peaks and introduce substantial angular deviation. In addition to the noise in the diffusion MRI, the additional isotropic component in our simulation model may corrupt the CSD results and lead to poor performance. The results of ball-and-sticks model show a similar pattern with CSD, but the accuracy is better in smaller crossing angles. Lastly, diffusion decomposition outperforms CSD and ball-and-sticks model in angles less than 45°, whereas in angles greater than 45°, its performance resembles the ball-and-sticks model. This feature can be attributed to the sparsity feature of the decomposition that offers good specificity while still retaining the sensitivity to crossing fibers.

Table 3 shows the result of a correlation analysis conducted to examine the accuracy of the fiber volume fractions provided by CSD, ball-and-sticks model, and diffusion decomposition. The analysis was conducted using the simulation dataset with 160 diffusion weighted images (SNR =  6.8).The correlation coefficients between the measured and simulated values are reported against different FA values. All methods provide good correlation (> 0.7), and higher FA values lead to higher correlation coefficients. Among these methods, ball-and-sticks model provides the best estimation of the fiber volume fraction, whereas diffusion decomposition slightly outperforms CSD.

**Table 3:** Correlation analysis on estimated fiber volume fraction.

FA	CSD	Ball-and-sticks	Decomposition
0.4	0.7783	0.8836	0.8109
0.5	0.8018	0.8984	0.8325
0.6	0.8226	0.9108	0.8461
0.7	0.8458	0.9215	0.8649

**Figure 3:**
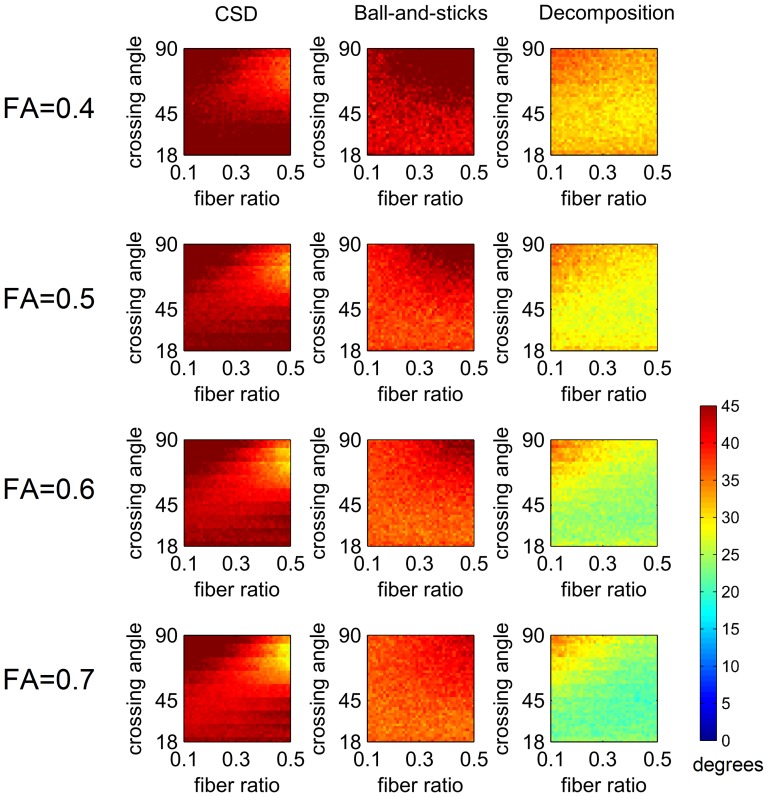
The average angular deviation of constrained spherical deconvolution (CSD), ball-and-sticks model, and diffusion decomposition applied to 55 diffusion weighted images with SNR of 2.3. Diffusion decomposition outperforms CSD and ball-and-sticks model in smaller crossing angles.

Figure 3 shows the average angular deviation of CSD, ball-and-sticks model, and diffusion decomposition applied to 55 diffusion weighted images with SNR of 2.3. All methods were applied to different combinations of crossing angle, fiber volume fractions, and FA. As shown in the figure, the CSD shows higher angular deviation in smaller crossing angles due to the false peaks, demonstrating its problem of specificity. By contrast, the results of the ball-and-sticks model show better accuracy in smaller crossing angles but not in larger crossing angles. The error may be due to model incompatibility (the ball-and-sticks model is a simplified model for multiple tensors), and the incompatibility error can be enhanced in low SNR conditions. Lastly, diffusion decomposition showed substantial better performance than CSD and ball-and-sticks model. The overall results suggest that diffusion decomposition can be applied to a diffusion dataset with 1) substantially reduced number of diffusion sampling and 2) low SNR.

### Phantom study

**Figure 4:**
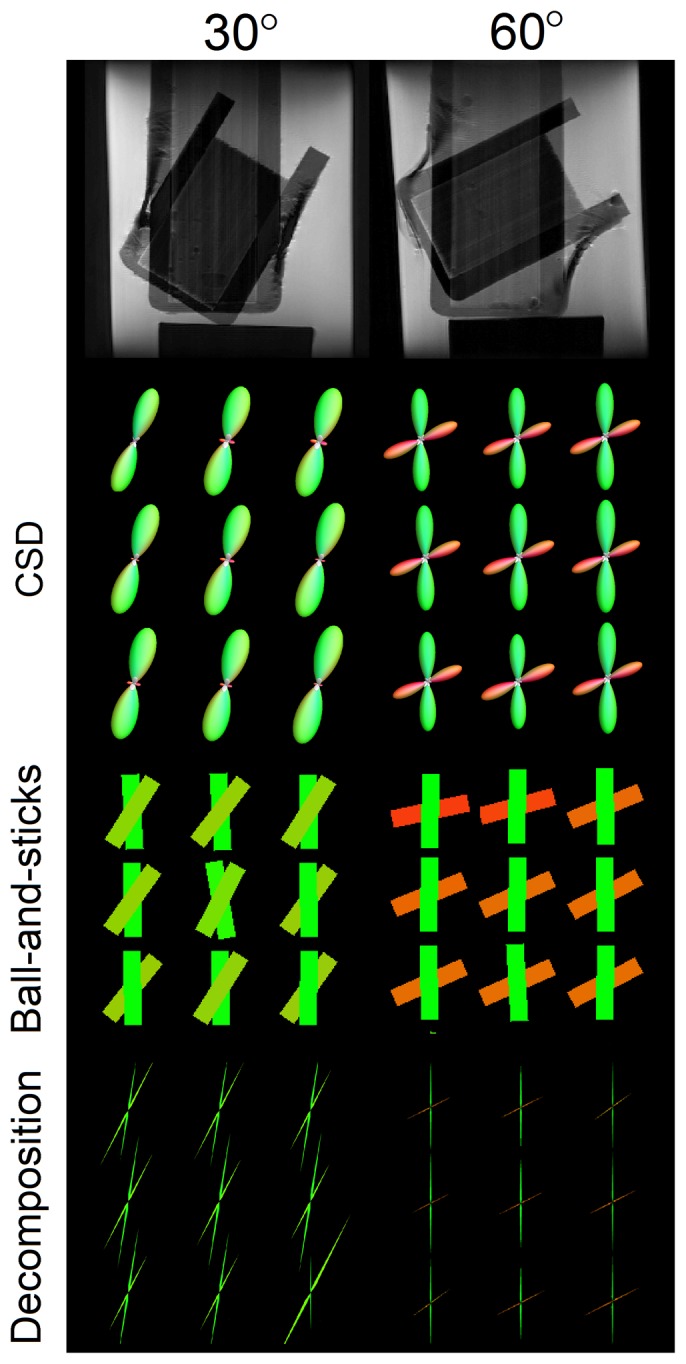
The result of constrained spherical deconvolution (CSD), ball-and-sticks model, and diffusion decomposition applied to phantom data. The results are from voxels at the center of the crossing region. All three methods resolve two fiber orientations at 60° crossing, but ball-and-sticks model present a slight tilting of the fiber orientations. At 30° crossing, CSD resolves one fiber orientation with several false fibers appeared around the origin due to baseline fluctuation, which is a typical specificity problem for deconvolution. By contrast, diffusion decomposition resolves two crossing fibers in all voxels at 30° crossing, and its fODF show positive value only at fiber orientations and zeros elsewhere, suggesting its sparsity feature.

Figure 4 shows the results of CSD, ball-and-sticks model, and diffusion decomposition applied to 30° and 60° crossing phantoms. The voxels at the center of the phantom are selected for visualization. All three methods are able to resolve crossing fibers at 60° crossing, however, ball-and-sticks model present a slight tilting of the fiber orientations. A possible cause to this error can be due to the discrepancy between the ball-and-sticks model and the actual diffusion pattern in the phantom. CSD and diffusion decomposition uses a consistent diffusion model (the response function was estimated from the phantom), and thus they do not have this drawback. At 30° crossing, CSD resolves one fiber orientation with several false fibers crossing at the horizontal direction. Ball-and-sticks model, despite the discrepancy of the diffusion model, is able to resolve two fibers. Diffusion decomposition method also resolves two crossing fibers and shows a clean baseline—positive values at fiber orientations and zeros elsewhere, underlining the sparsity feature of the decomposition algorithm. This is in contrast to CSD, which has fluctuation around the origin that may give rise to false fibers and compromise its specificity.

**Figure 5:**
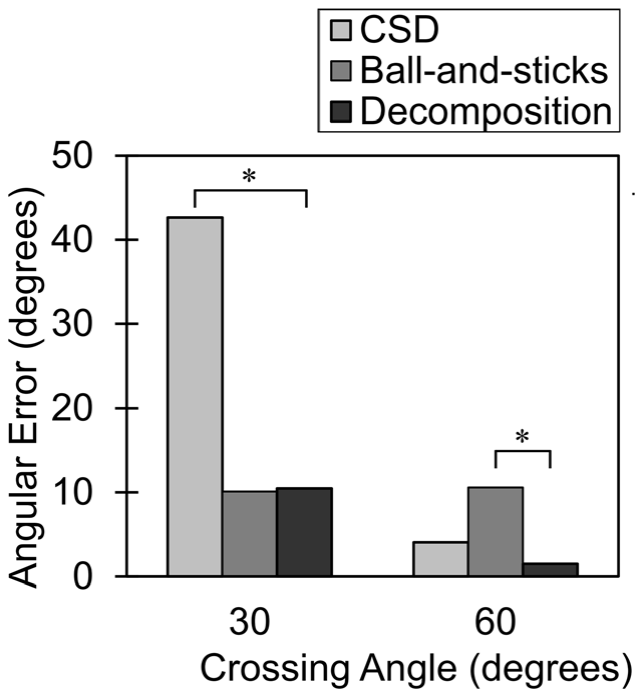
The angular error of constrained spherical deconvolution (CSD), ball-and-sticks model, and diffusion decomposition applied to phantom data. The diffusion decomposition shows significantly better accuracy than CSD at 30° crossing and ball-and-sticks model at 60° crossing (p-value < 0.001).

Figure 5 shows the angular error of CSD, ball-and-sticks model, and diffusion decomposition applied to 30° and 60° crossing phantom. At 60°crossing, both CSD and diffusion decomposition show angular deviations lower than 10°, while ball-and-sticks model has a significant increase (p < 0.001) in angular deviation. At 30° crossing, ball-and-sticks model and diffusion decomposition shows similar angular deviations around 10°, while CSD has a significant increase (p < 0.001) in the angular error due to false fibers resolved at the horizontal directions.

### In-vivo study

**Figure 6:**
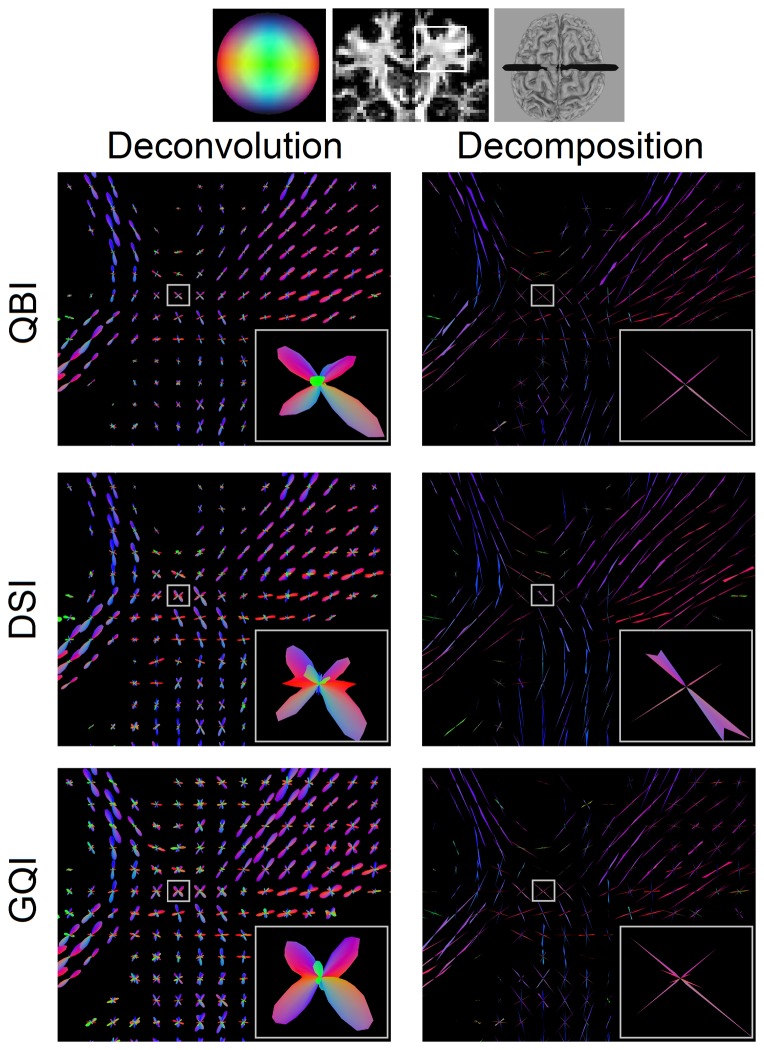
The fiber ODFs obtained by applying diffusion deconvolution and diffusion decomposition to QBI, DSI, and GQI. Each of the methods reconstructs diffusion ODF from shell, grid, and two-shell sampling schemes, respectively. Both diffusion deconvolution and diffusion decomposition can be equally applied to QBI, DSI, and GQI to improve their ability to resolve crossing fibers, but the fiber ODFs obtained from deconvolution shows blunt lobes with fluctuating baseline, whereas those from decomposition show sharp spikes with clean baseline, suggesting that diffusion decomposition can achieve better sensitivity and specificity in resolving crossing fibers.

Figure 6 shows the fODFs obtained by applying diffusion deconvolution and diffusion decomposition to the dODFs acquired by DSI, QBI, and GQI. The figure is presented at the coronal view of centrum semiovale. Both diffusion deconvolution and diffusion decomposition are applicable to different q-space imaging methods and different diffusion sampling schemes, and the resulting fODFs show consistent fiber orientations. However, the fODFs estimated by the decomposition method show sharp spikes with a clean baseline owing to the sparsity feature. In contrast, the fODFs estimated by the deconvolution method show blunt lobes with a fluctuated baseline. The above comparison suggests that diffusion decomposition can achieve better angular resolution and specificity in the *in-vivo* study.

**Figure 7:**
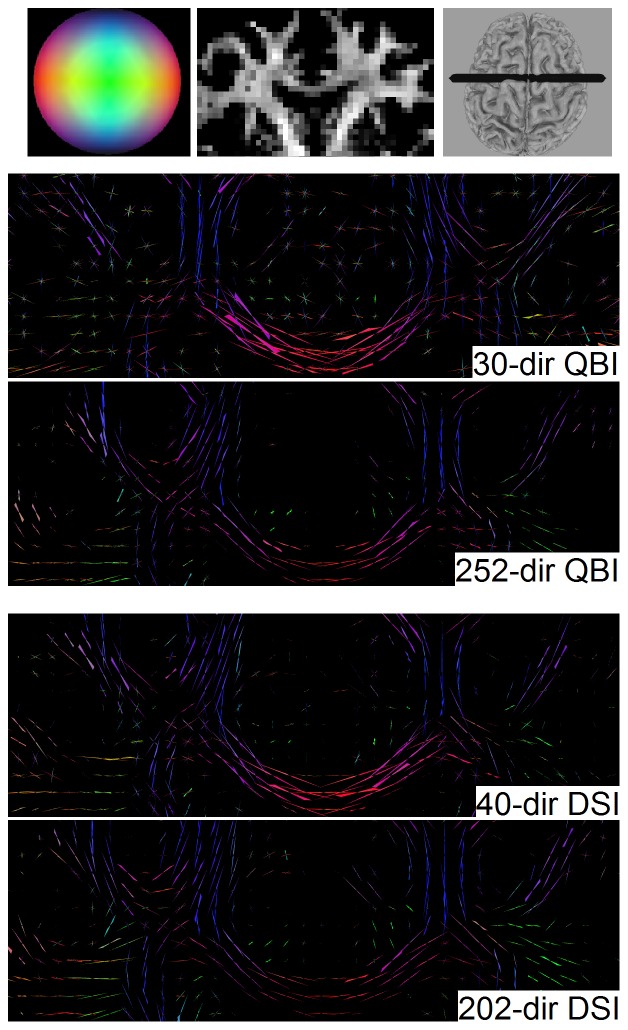
The fiber ODFs obtained from diffusion decomposition applied to the 30-direction QBI, 252-direction QBI, 40-direction DSI, and 202-direction DSI. Even in datasets with substantially reduced number of diffusion encoding directions, diffusion decomposition can still depict correct crossing fibers formed by the corpus callosum and corticospinal tract.

Figure 7 shows the fODFs obtained from diffusion decomposition applied to the 30-direction QBI, 252-direction QBI, 40-direction DSI, and 202-direction DSI. The figure presents the centrum semiovale in the coronal view. As shown in Fig. 7, diffusion decomposition can reveal crossing fibers formed by the corpus callosum (horizontal fibers) and corticospinal tracts (vertical fibers) using reduced sampling schemes, and the crossing fiber orientations are consistent with those of the full schemes. The specificity of diffusion decomposition can be appreciated in the mid corpus callosum, where it presents single fiber population with correct orientation and is free from false fibers. The above results suggest that diffusion decomposition can be applied to a reduced dataset to provide correct information about fiber orientations.

**Figure 8:**
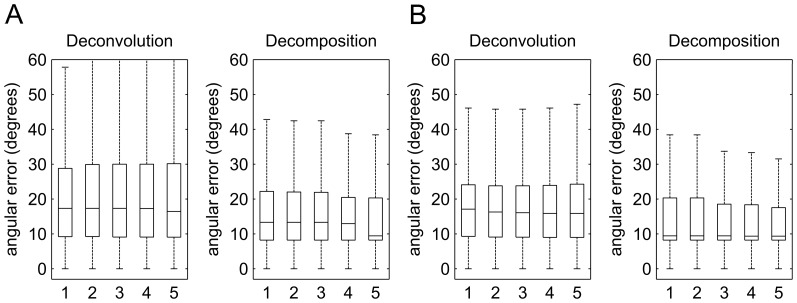
The boxplot showing the distribution of the angular error related to sensitivity. The results from (A) 30-direction QBI and (B) 40-direction DSI are shown in the figure. Diffusion deconvolution and diffusion decomposition was examined by 5 different parameters. The result shows that the decomposition approach presents lower values in the medians and upper quartiles than those of diffusion deconvolution, suggesting that diffusion decomposition is more sensitive than deconvolution.

Figure 8 shows the boxplot of the angular error related to sensitivity. Figure 8A shows the results obtained from the 30-direction QBI, whereas Figure 8B shows the results obtained from the 40-direction DSI. Both diffusion deconvolution and diffusion decomposition were tested using 5 different parameters to compare their performance. In both QBI and DSI, diffusion decomposition shows lower values in medians and upper quartiles regardless of the parameter settings applied, suggesting that diffusion decomposition is more sensitive to crossing.

**Figure 9:**
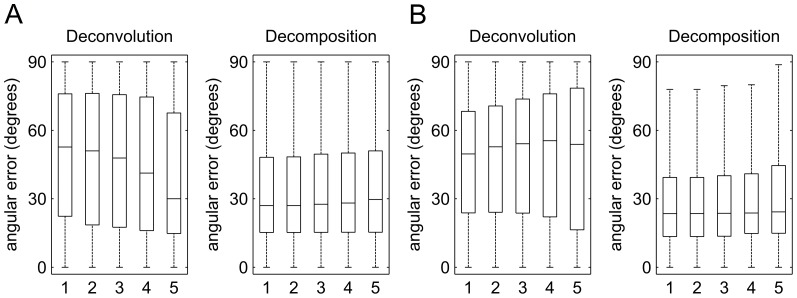
The boxplot showing the distribution of the angular error related to the specificity. The results from (A) 30-direction QBI and (B) 40-direction DSI are shown in the figure. Diffusion deconvolution and diffusion decomposition was examined by 5 different parameters. The result shows that the decomposition approach presents lower values in the medians and upper quartiles than those of diffusion deconvolution, suggesting that the result obtained from diffusion decomposition is more specific.

Figure 9 shows the boxplot of the angular error related to specificity. Figure 9A shows the results obtained from the 30-direction QBI, whereas Figure 9B shows the results obtained from the 40-direction DSI. In both datasets, diffusion decomposition shows substantially lower values in medians and upper quartiles, regardless of the parameter settings. This specificity study suggests that the fibers resolved by diffusion decomposition are more specific than those by diffusion convolution.

Table 4 lists the mean angular error of diffusion deconvolution and diffusion decomposition in the sensitivity and specificity tests. In the sensitivity test, the mean angular errors of diffusion deconvolution are around 20° in 30-direction QBI and 18° in 40-direction DSI, whereas those of the decomposition method are around 16° in both 30-direction QBI and 40-direction DSI. The decomposition method has an average of 4.87° less angular error in 30-direction QBI and 2.39° less angular error in 40-direction DSI. The difference is statistically significant (p-value < 0.001). Similarly, the specificity test demonstrates consistent conclusion but with a more substantial difference. The mean angular errors of diffusion deconvolution range from 38° to 49° in 30-direction QBI and 47° to 49° in 40-direction DSI, whereas those of the decomposition method are around 33° in 30-direction QBI and 29° in 40-direction DSI. The decomposition method has an average of 11.78° less angular error in 30-direction QBI and 19.20° less angular error in 40-direction DSI. The difference is statistically significant (p-value < 0.001). In terms of sensitivity and specificity, diffusion decomposition achieved significantly better performance than diffusion deconvolution.

**Table 4:** Mean angular error of diffusion deconvolution and diffusion decomposition in 30-direction QBI and 40-direction DSI.

Sensitivity test	Dataset	Results under different parameters^a^				
Diffusion deconvolution	30-dir QBI	20.19°	20.60°	20.77°	21.02°	22.12°
	40-dir DSI	18.36°	17.98°	17.73°	17.84°	18.33°
Diffusion decomposition	30-dir QBI	16.39°	16.29°	16.08°	15.90°	15.68°
	40-dir DSI	16.11°	16.04°	15.80°	15.45°	14.87°
**Specificity test**						
Diffusion deconvolution	30-dir QBI	49.02°	48.33°	47.20°	44.67°	38.75°
	40-dir DSI	47.12°	48.62°	49.54°	49.69°	48.61°
Diffusion decomposition	30-dir QBI	33.35°	33.44°	33.73°	34.02°	34.51°
	40-dir DSI	28.73°	28.86°	29.23°	29.85°	30.89°

a Diffusion deconvolution was conducted by regularization parameters of 1, 2, 4, 8, and 16. Diffusion decomposition was conducted by decomposition fractions of 0.01, 0.02, 0.05, 0.1, and 0.2.

**Figure 10:**
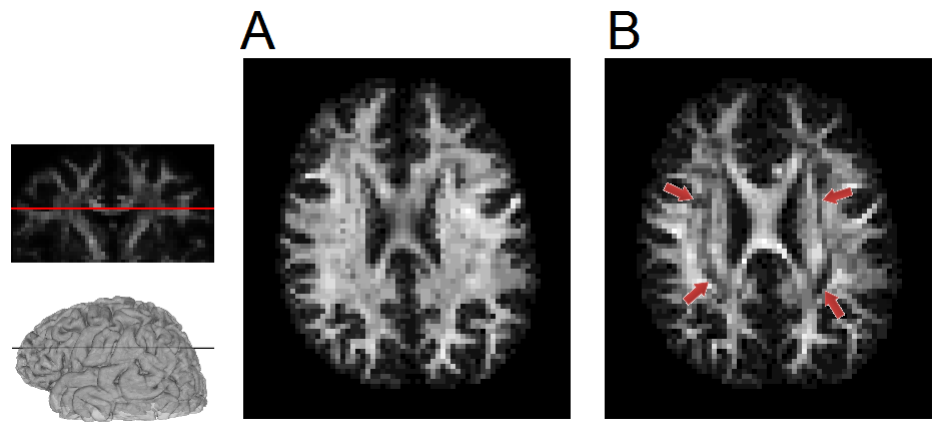
Fiber volume mapping versus FA mapping. (A) The mapping of the total fiber volume calculated from diffusion decomposition and (B) the mapping of the fractional anisotropy (FA) calculated from the diffusion tensor analysis applied to the same data. The FA mapping shows decreased values in the centrum semiovale due to the crossing fibers in this region (annotated), whereas the total fiber volume mapping shows a relatively homogeneous intensity throughout the white matter. This suggests that the fiber volume can provide a better gray-white matter separation and facilitate further investigation into structural integrity.

Figure 10 shows the mapping of the total fiber volume fraction derived from diffusion decomposition (Fig. 10A) and the mapping of FA (Fig. 10B). The total fiber volume fraction was calculated by summing all fiber volume fractions in our mixed diffusion model (*f*_0_ excluded), whereas the FA was calculated from the tensor analysis. Both images were obtained from the same 202-direction DSI dataset in an axial view at the level of the corpus callosum. The intensities of the images were scaled by their relative contrast so that the maximum value corresponded to the maximum intensity. Although the total fiber volume fraction mapping shows a pattern similar to the FA mapping, decreased values of FA can be observed in the regions where the corpus callosum crosses the corticospinal tracts (indicated by arrows). By contrast, the total fiber volume fraction mapping shows a relative homogeneous distribution of the intensity throughout the white matter. The above comparison suggests that the total fiber volume fraction is less affected by crossing fibers and may serve as an index specific to fiber volume fraction of white matter tracts.

## Discussion

This paper proposes a sparse fODF estimation method called diffusion decomposition, which obtains fODF by decomposing the dODF acquired from DSI, QBI, or GQI. The simulation study shows that diffusion decomposition offers better accuracy than CSD and ball-and-sticks model in the dataset with substantially reduced number of diffusion sampling directions. The phantom study shows that the fODF of diffusion decomposition presents the sparsity feature—zero values at most orientations and positive values only at fiber orientations. This is in contrast to the fODF of CSD, which presents fluctuation at the baseline and often requires filtering to eliminate false fibers. Further quantitative analysis shows that diffusion decomposition presents significantly lower angular error than that of CSD at 30° crossing or ball-and-sticks model at 60° crossing. Similar conclusion can be drawn from the results in the *in-vivo* study where diffusion decomposition outperforms diffusion deconvolution owing to the sparsity feature of the solution.

From the perspective of computation, diffusion decomposition seems to be a hybrid of the model-fitting (e.g. ball-and-sticks model) and deconvolution approach. The performance in resolving crossing fibers is improved by promoting sparsity. Compared with model-fitting approach such as ball-and-sticks model, an interesting finding in our study is that while diffusion decomposition presents similar performance in both simulation and phantom study, ball-and-sticks model seems to perform worse in the phantom at 60° crossing than it does in the simulation. One possible reason for this underperformance may be due to the poor fitting of ball-and-sticks with the actual diffusion model in the phantom study. It is possible that the diffusion model is more complicated in real world scenario, and ball-and-sticks may not be adequate for fitting its signal responses. Compared with deconvolution, the outperformance of diffusion decomposition can be summarized in two aspects: sensitivity and specificity. The increased sensitivity can be attributed to the spiky fODF obtained by *L*_1_ regularization, whereas the fODF obtained by *L*_2_ regularization still retains part of the dODF blurring that leads to less sensitive results. The second aspect of the outperformance—specificity—is even more substantial. While the angular error related to sensitivity is improved by ∼3° in the reduced datasets, almost negligible in terms of the ODF angular resolution, the angular error related to specificity is improved by ∼15°. The substantial improvement in the specificity can be expected. As we have pointed out in our previous deconvolution study [11], the deconvolution method does not guarantee a good specificity although it has been shown to achieve a good sensitivity to crossing fibers. Higher smoothing or ODF filtering is often required to remove false fibers, but less salient fibers will also be removed indiscriminately. By contrast, diffusion decomposition offers a better specificity owing to the sparsity nature of *L*_1_ regularization. The clean baseline of the sparse fODFs can avoid false identification of the crossing fibers. One should note that this outperformance does not come without a price. Diffusion decomposition sacrifices smoothness for sparsity to obtain better resolving power. Consequently, it cannot portray fiber dispersion due to the sparsity assumption. Nonetheless, since the fiber dispersion phenomenon is more obvious in low spatial resolution images, it may be possible to mitigate this limitation by acquiring diffusion images at a higher spatial resolution. Further study may be needed to fully investigate this limitation under different resolutions.

There are several other methods that have promoted sparsity in the spherical harmonics, whereas we promoted it in the ODF space. The advantage of our approach is that a sparse fODF can be easily represented by an ODF vector but *not* by spherical harmonics. This can be readily examined by calculating the spherical harmonics of a delta function (i.e. the fODF of a single fiber). It results in infinite non-zero responses of spherical harmonics, meaning that one cannot use a finite order of spherical harmonics to represent a single-fiber fODF. Methods based on spherical harmonics inevitably truncate higher responses of the spherical harmonics, and their estimated fODF becomes a smoothed version of the true fODF. Consequently, enforcing sparsity in the ODF space may achieve better accuracy.

In addition to the outperformance, another noteworthy feature of diffusion decomposition is its ability to resolve crossing fibers based on 30-direction QBI and 40-direction DSI. This feature is particularly important since QBI and DSI have long been criticized for lengthy scanning time that limits their applications in clinical study. As shown in an optimization study [31], QBI and DSI typically requires maximal diffusion sensitivity about 3000 to 4000 s/mm^2^ and more than 200 diffusion encoding directions, resulting in a scanning time of approximately 30 min. In this study, the 30-direction QBI was acquired using the built-in DTI sequence in the clinical scanner, which is readily available for the most of the MRI facilities. The 40-drection DSI needs only a maximum b-value of 2300 sec/mm^2^, a setting that greatly reduces the demand for the gradient performance. For either 30-direction QBI or 40-direction DSI, the scanning time of the diffusion acquisition is approximately 5 minutes, making it a realistic option for inclusion in clinical studies. Nevertheless, one should note that the reduction of the number of gradient directions still has its shortcoming. Although diffusion decomposition can resolve crossing fibers in the white matter, we observe that its sensitivity is compromised at the gray-white matter junction. This could be due to the low SNR of diffusion MR signals in the region, and there seems to be a trade-off between the SNR and the number of diffusion gradient directions.

Lastly, diffusion decomposition has other features that are also worth mentioning. 1) It is equally applicable to grid, single-shell, and multi-shell encoding schemes. This makes the algorithm a convenient tool for data with various combinations of b-values and diffusion encoding schemes. 2) The fiber volume fraction is assigned to a specific fiber orientation, and thus it can be used as an index to quantify structural connectivity for human connectome study. The total fiber volume fraction derived from the decomposition method can also be used to delineate the gray-white matter junction and may be used to examine structural integrity.

There are drawbacks in diffusion decomposition. Similar to deconvolution methods, diffusion decomposition uses a putative common characteristic dODF to model the single fiber direction compartment despite the fact that the characteristic dODF varies across different regions in the white matter. As shown in our previous study, the dODFs obtained from the corpus callosum, cingulum bundle, and corticospinal tracts have different profiles [11]. Applying a universal characteristic ODF to fit all scenarios may introduce additional errors, and this drawback has been reported to decrease the specificity of spherical deconvolution [18]. Although this problem was not rigorously examined in this study, we believe that our method is also susceptible to this drawback as long as a universal characteristic dODF is used. Possible improvement may be achieved by using multiple characteristic dODFs instead of a common dODF or using an unsupervised clustering approach to characterize the component dODFs. Furthermore, the 642-direction ODF has a limited angular resolution around 8 degrees, and diffusion decomposition therefore has an inherent error due to limited resolution of an ODF. Lastly, a fixed value of decomposition volume was used for all voxels in our study. In practice, it is possible to optimize this value for each voxel using cross-validation, and this is a potential future work to further improve the performance of diffusion decomposition.

In conclusion, diffusion decomposition has a particular role in diffusion datasets with substantially reduced number of diffusion encoding directions, making it highly feasible for clinical studies that allows a limited scanning time. The decomposition algorithm provides a sparse solution of fODF to improve the ability in resolving crossing fibers and to avoid false fibers as encountered in diffusion deconvolution. The algorithm can be applied to diffusion data and to facilitate further fiber tracking algorithms. These features may be valuable for future human connectome study to define brain connectivity.

## References

[1] Akil H, Martone ME, Van Essen DC (2011) Challenges and opportunities in mining neuroscience data. Science 331: 708–712. 21311009 10.1126/science.1199305 PMC3102049

[2] Ciccarelli O, Catani M, Johansen-Berg H, Clark C, Thompson A (2008) Diffusion-based tractography in neurological disorders: concepts, applications, and future developments. Lancet Neurol 7: 715–727. 18635020 10.1016/S1474-4422(08)70163-7

[3] Jones DK, Knosche TR, Turner R (2013) White matter integrity, fiber count, and other fallacies: The do's and don'ts of diffusion MRI. Neuroimage 73: 239–254. 22846632 10.1016/j.neuroimage.2012.06.081

[4] Tuch DS (2004) Q-ball imaging. Magn Reson Med 52: 1358–1372. 15562495 10.1002/mrm.20279

[5] Tuch DS, Reese TG, Wiegell MR, Makris N, Belliveau JW, et al (2002) High angular resolution diffusion imaging reveals intravoxel white matter fiber heterogeneity. Magn Reson Med 48: 577–582. 12353272 10.1002/mrm.10268

[6] Wedeen VJ, Hagmann P, Tseng WY, Reese TG, Weisskoff RM (2005) Mapping complex tissue architecture with diffusion spectrum magnetic resonance imaging. Magn Reson Med 54: 1377–1386. 16247738 10.1002/mrm.20642

[7] Yeh FC, Wedeen VJ, Tseng WY (2010) Generalized q-sampling imaging. IEEE Trans Med Imaging 29: 1626–1635. 20304721 10.1109/TMI.2010.2045126

[8] Tournier JD, Calamante F, Gadian DG, Connelly A (2004) Direct estimation of the fiber orientation density function from diffusion-weighted MRI data using spherical deconvolution. Neuroimage 23: 1176–1185. 15528117 10.1016/j.neuroimage.2004.07.037

[9] Tournier JD, Yeh CH, Calamante F, Cho KH, Connelly A, et al (2008) Resolving crossing fibres using constrained spherical deconvolution: Validation using diffusion-weighted imaging phantom data. Neuroimage 42: 617–625. 18583153 10.1016/j.neuroimage.2008.05.002

[10] Dell'acqua F, Scifo P, Rizzo G, Catani M, Simmons A, et al (2009) A modified damped Richardson-Lucy algorithm to reduce isotropic background effects in spherical deconvolution. Neuroimage 49: 1446–1458. 19781650 10.1016/j.neuroimage.2009.09.033

[11] Yeh FC, Wedeen VJ, Tseng WY (2011) Estimation of fiber orientation and spin density distribution by diffusion deconvolution. Neuroimage 55: 1054–1062. 21232611 10.1016/j.neuroimage.2010.11.087

[12] Kezele I, Descoteaux M, Poupon C, Poupon F, Mangin JF (2010) Spherical wavelet transform for ODF sharpening. Med Image Anal 14: 332–342. 20207188 10.1016/j.media.2010.01.002

[13] Patel V, Shi Y, Thompson PM, Toga AW (2010) Mesh-based spherical deconvolution: a flexible approach to reconstruction of non-negative fiber orientation distributions. Neuroimage 51: 1071–1081. 20206705 10.1016/j.neuroimage.2010.02.060 PMC2927199

[14] Alexander DC (2005) Maximum entropy spherical deconvolution for diffusion MRI. Inf Process Med Imaging 19: 76–87. 17354686 10.1007/11505730_7

[15] Kaden E, Kruggel F (2012) Nonparametric Bayesian inference of the fiber orientation distribution from diffusion-weighted MR images. Med Image Anal 16: 876–888. 22381587 10.1016/j.media.2012.01.004

[16] Schultz T, Westin CF, Kindlmann G (2010) Multi-diffusion-tensor fitting via spherical deconvolution: a unifying framework. Med Image Comput Comput Assist Interv 13: 674–681. 20879289 10.1007/978-3-642-15705-9_82 PMC4739653

[17] Ramirez-Manzanares A, Rivera M, Vemuri BC, Carney P, Mareci T (2007) Diffusion basis functions decomposition for estimating white matter intravoxel fiber geometry. IEEE Trans Med Imaging 26: 1091–1102. 17695129 10.1109/TMI.2007.900461

[18] Parker GD, Marshall AD, Rosin PL, Drage N, Richmond S, et al (2013) A pitfall in the reconstruction of fibre ODFs using spherical deconvolution of diffusion MRI data. Neuroimage 65: 433–448. 23085109 10.1016/j.neuroimage.2012.10.022 PMC3580290

[19] Landman BA, Bogovic JA, Wan H, El Zahraa ElShahaby F, Bazin PL, et al (2012) Resolution of crossing fibers with constrained compressed sensing using diffusion tensor MRI. Neuroimage 59: 2175–2186. 22019877 10.1016/j.neuroimage.2011.10.011 PMC3254826

[20] Hesterberg T, Choi NH, Meier L, Fraley C (2008) Least angle and ℓ1 penalized regression: A review. Statist Surv 2: 61–93.

[21] Tibshirani R (1996) Regression shrinkage and selection via the lasso. J Royal Statist Soc B 58: 267–288.

[22] Efron B, Hastie T, Johnstone I, Tibshirani R (2004) Least angle regression. The Annals of Statistics 32: 407–499.

[23] Hastie T, Taylor J, Tibshirani R, Walther G (2007) Forward stagewise regression and the monotone lasso. Electron J Statist 1: 1–29.

[24] Tournier JD, Calamante F, Connelly A (2007) Robust determination of the fibre orientation distribution in diffusion MRI: non-negativity constrained super-resolved spherical deconvolution. Neuroimage 35: 1459–1472. 17379540 10.1016/j.neuroimage.2007.02.016

[25] Behrens TE, Woolrich MW, Jenkinson M, Johansen-Berg H, Nunes RG, et al (2003) Characterization and propagation of uncertainty in diffusion-weighted MR imaging. Magn Reson Med 50: 1077–1088. 14587019 10.1002/mrm.10609

[26] Descoteaux M, Deriche R, Knosche TR, Anwander A (2009) Deterministic and probabilistic tractography based on complex fibre orientation distributions. IEEE Trans Med Imaging 28: 269–286. 19188114 10.1109/TMI.2008.2004424

[27] Jeurissen B, Leemans A, Tournier JD, Jones DK, Sijbers J (2012) Investigating the prevalence of complex fiber configurations in white matter tissue with diffusion magnetic resonance imaging. Hum Brain Mapp: May 19 [Epub ahead of print]. 10.1002/hbm.22099 PMC6870534 22611035

[28] Wedeen VJ, Rosene DL, Wang R, Dai G, Mortazavi F, et al (2012) The geometric structure of the brain fiber pathways. Science 335: 1628–1634. 22461612 10.1126/science.1215280 PMC3773464

[29] Alexander DC, Barker GJ, Arridge SR (2002) Detection and modeling of non-Gaussian apparent diffusion coefficient profiles in human brain data. Magn Reson Med 48: 331–340. 12210942 10.1002/mrm.10209

[30] Gudbjartsson H, Patz S (1995) The Rician distribution of noisy MRI data. Magn Reson Med 34: 910–914. 8598820 10.1002/mrm.1910340618 PMC2254141

[31] Kuo LW, Chen JH, Wedeen VJ, Tseng WY (2008) Optimization of diffusion spectrum imaging and q-ball imaging on clinical MRI system. Neuroimage 41: 7–18. 18387822 10.1016/j.neuroimage.2008.02.016

